# Atherosclerosis of the descending aorta predicts cardiovascular events: a transesophageal echocardiography study

**DOI:** 10.1186/1476-7120-2-21

**Published:** 2004-10-22

**Authors:** Albert Varga, Noemi Gruber, Tamás Forster, Györgyi Piros, Kálmán Havasi, Éva Jebelovszki, Miklos Csanády

**Affiliations:** 1 2^nd ^Department of Medicine and Cardiology Center, University of Sciences, Szeged, Hungary

## Abstract

**Purpose:**

Previous studies have shown that atherosclerosis of the descending aorta detected by transesophageal echocardiography (TEE) is a good marker of coexisting coronary artery disease. The aim of our study was to evaluate whether the presence of atherosclerosis on the descending aorta during TEE has any prognostic impact in predicting cardiovascular events.

**Material and Methods:**

The study group consisted of 238 consecutive in-hospital patients referred for TEE testing (135 males, 103 females, mean age 58 +/- 11 years) with a follow up of 24 months. The atherosclerotic lesions of the descending aorta were scored from 0 (no atherosclerosis) to 3 (plaque >5 mm and/or "complex" plaque with ulcerated or mobile parts).

**Results:**

Atherosclerosis was observed in 102 patients, (grade 3 in 16, and grade 2 in 86 patients) whereas 136 patients only had an intimal thickening or normal intimal surface. There were 57 cardiovascular events in the follow-up period. The number of events was higher in the 102 patients with (n = 34) than in the 136 patients without atherosclerosis (n = 23, p < 0.01). The frequency of events was in close correlation with the severity of the atherosclerosis of the descending aorta. Fifty percent of the patients with grade 3 experienced cardiovascular events. Excluding patients with subsequent revascularization, the multivariate analysis only left ventricular function with EF < 40% (HR 3.0, CI 1.3–7.1) and TEE atherosclerotic plaque >=2 (HR 2.4, CI 1.0–5.5) predicted hard cardiovascular events.

**Conclusion:**

Atherosclerosis of the descending aorta observed during transesophageal echocardiography is a useful predictor of cardiovascular events.

## Introduction

During the past decades many methods and factors have been proposed as good prognostic tools or markers for cardiovascular risk stratification. However, even with the most sophisticated stress testing procedures the prediction capability still remains imperfect [1]. Previous studies have shown that atherosclerosis of the descending aorta detected by transesophageal echocardiography (TEE) is a good marker of coexisting coronary artery disease [2-9]. Cohen et al. have demonstrated, that in patients with brain infarction, "complex" plaques (with ulcerated surface, mobile parts and thrombi) are powerful predictors of future cardiovascular events [10]. The aim of this study was to evaluate whether the presence of atherosclerosis on the descending aorta observed by routine scanning during TEE has any prognostic impact in predicting cardiovascular events such as cardiac death, myocardial infarction or fatal stroke. Therefore, we conducted a prospective study; collecting and analyzing the data of 238 consecutive patients referred to the echo lab for transesophageal echocardiography.

## Methods

### Patient selection

During the year 1998, 238 consecutive patients (135 males, 103 females, and mean age 58 ± 11 years) were studied with transesophageal echocardiography at the 2^nd ^Department of Medicine and Cardiology Center, University of Sciences, Szeged, Hungary. The patients underwent TEE examination for the following reasons: TIA or suspected cerebral embolism (n = 100), coronary flow reserve evaluation (n = 71), evaluation of the native or artificial mitral valve (n = 23), suspected endocarditis (n = 15), evaluation of the aortic valve (n = 13), suspected aortic dissection (n = 6), atrial septal defect or patent foramen ovale (n = 6), others (n = 4). Thirty patients had suffered a previous myocardial infarction. The patients were followed-up to a period of at least 2 years, median 31 ± 9 months.

### Transthoracic and transesophageal echocardiography

All patients had a transthoracic echocardiographic examination to assess the global and regional left ventricular function. The ejection fraction was calculated using the area-length single plane method [11]. Left ventricular function was considered to be depressed in case of ejection fraction ≤40%.

The TEE examination was carried out according to recommendations of the Mayo clinic procedure [12]. Two-dimensional echocardiograms were obtained by using commercially available imaging system (ATL-HDI) with a biplane transducer. Echocardiographic images were recorded on videotape for subsequent playback and analysis. The atherosclerotic lesions of the descending aorta were graded according to the modified scoring system originally proposed by Fazio et al [2]:

Grade 0 – no sign of atherosclerosis

Grade 1 – intimal thickening

Grade 2 – plaque < 5 mm

Grade 3 – plaque > 5 mm and/or "complex" plaque with ulcerated or mobile parts.

**Figure 1:**
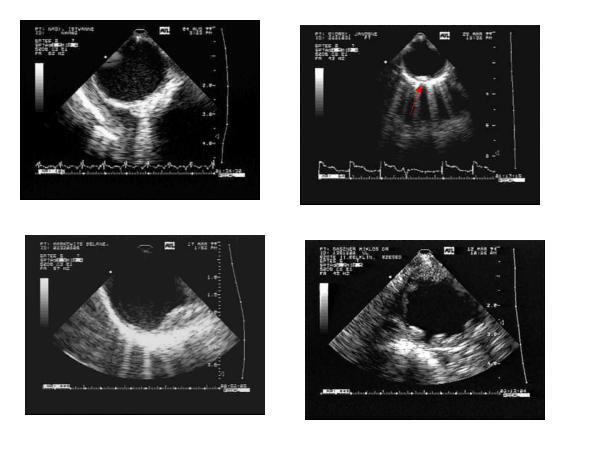
Grading of the transesophageally detected aortic lesions in the descending aorta. Grade 1: intimal thickening (left upper panel); Grade 2: small plaque indicated by arrow right upper panel; Grade 3: lower panels. On the lower panels: left a huge, multiple plaque, right: an ulcerated plaque with mobile part.

Significant atherosclerosis was considered in case of grade 2 or 3 (fig 1 and additional files 1, 2, 3, 4).

### Follow up data

Follow-up data were obtained from at least one of four sources: 1) review of the patient's hospital record; 2) personal communication with the patient's physician and review of the patient's chart; 3) a telephone interview with the patient conducted by trained personnel; or 4) a staff physician visiting the patients at regular intervals in the outpatient clinic. Follow-up data were obtained in all patients. The outcome events were: cardiac related death, fatal stroke, non-fatal myocardial infarction and revascularization either by percutaneous transluminal coronary angioplasty (PTCA) or by coronary artery by-pass grafting (CABG). Myocardial infarction was documented by a consistent history, EKG changes and cardiac enzyme level elevations and confirmed by hospital chart or hospital discharge letter review. In the next step, patients with revascularization were censored and the remaining patients were considered for the analysis of hard events (cardiac death, myocardial infarction, and fatal stroke). All-cause mortality (cardiovascular death plus death of other causes) was also analyzed.

### Statistical analysis

Values are expressed as mean ± Standard Deviation. Continuous variables have been compared by the means of Student's t test (two-tailed). Statistical analysis of discrete variables has been performed with chi-square test; a Fisher's exact test has been used when appropriate. The individual effect of certain variables on infarction-free survival has been evaluated with the use of the Cox proportional hazard model with univariate and multivariate analysis and with the Kaplan-Meier method. The patients were stratified into two subgroups: patients with and without cardiac events. The examined variables were age, sex, risk factors (arterial hypertension, diabetes, hypercholesterolemia), previous myocardial infarction, ejection fraction, and significant atherosclerosis of the descending aorta. A p value <0.05 was considered statistically significant.

## Results

### Transesophageal echocardiography

The examination was successful and complete in all patients, without any side effect. Significant atherosclerosis of the descending aorta was observed in 102 patients, (grade 3 in 16, and grade 2 in 86 patients) whereas 136 patients had only mild intimal thickening (n = 46) or normal endocardial surface (n = 90).

### Follow-up data

There were 57 events in the follow-up period: cardiac related death: n = 14, fatal stroke: n = 4, non-fatal myocardial infarction: n = 5 and coronary artery revascularization: n = 34. Ten patients died of non-cardiovascular causes and the cause of the death of 2 patients was undetermined.

### Cardiovascular events and TEE findings

The number of events was significantly higher in the 102 patients with (n = 34) as in patients the 136 patients without significant atherosclerosis (n = 23, p < 0.01). The results of univariate and multivariate analysis are shown in table 1 and 2.

**Table 1:** Univariate analysis – all events (cardiac death + nonfatal AMI + stroke + revascularization)

**Variable**	**p value**
Aorta plaque	0,0033
Previous MI	0,0074
Male gender	0,0088
Hypertension	0,0758
Age	0,1687
Cholesterol	0,1154
Diabetes	0,2954
LV EF	0,7810

LV EF = Left ventricular ejection fraction; MI = myocardial infarction

**Table 2:** Multivariate analysis – all events (cardiac death + nonfatal AMI + stroke + revascularization)

			**95% CI**	
**Variable**	**p value**	**HR**	**Lower**	**Upper**

Aorta plaque	<0,01	2,1	1,2	3,5
Male gender	<0,05	0,49	0,3	0,9

HR = Hazard Ratio; CI = Confidence Interval

**Figure 2:**
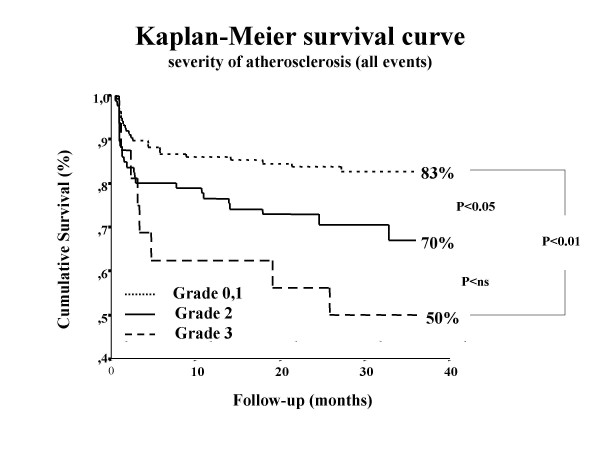
A Kaplan-Meier curve showing the association between the severity of the transesophageally detected aortic plaques and the long term survival including all events. It can be clearly seen that more severe the atherosclerosis on the descending aorta was, higher the probability of future cardiovascular events.

The frequency of events was in close correlation with the severity of the atherosclerosis of the descending aorta (fig 2). Fifty percent of the patients with grade 3 experienced cardiovascular events. There was no significant difference when all causes of death were considered between subjects with aortic lesions or free of atherosclerosis of the descending aorta (5% vs 1%, p = ns).

### Spontaneous cardiovascular events and TEE findings

Patients with early (<3 months, n = 29) or late (>3 months, n = 5) revascularization have been censored.

**Figure 3:**
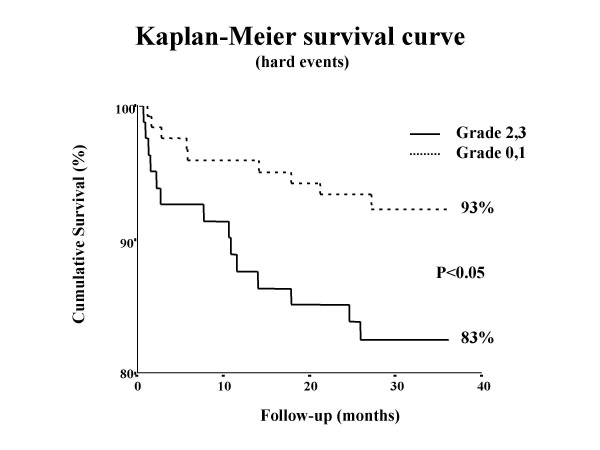
A Kaplan-Meier curve survival showing a better outcome of patients without transesophageally detected aortic plaques.

Nine events have occurred in the 122 patients with TEE score ≤1 and 14 in the 82 patients with TEE score ≥2 (7% vs 17%, p < 0.05) (fig 3). The results of univariate analysis are shown in table 3. Impaired left ventricular function (EF ≤ 40%), significant atherosclerosis of the descending aorta, and age were predictive for future cardiovascular events. By multivariate analysis, only left ventricular function with EF ≤ 40% (HR 3.0, 95% CI 1.3–7.1) and TEE atherosclerotic plaque ≥2 (HR 2.4, 95% CI 1.0–5.5) predicted cardiovascular events (table 4). Similarly to the entire group, in patients with no revascularization, the more severe the atherosclerosis on the descending aorta was, higher the probability of future cardiovascular events. There was no significant difference when all causes of death were considered between subjects with aortic lesions or free of atherosclerosis of the descending aorta (5% vs 1%, p = ns).

**Table 3:** Univariate analysis – Hard events (cardiac death + nonfatal AMI + stroke)

**Variable**	**p value**
LV EF	0,0069
Aorta plaque	0,0283
Age	0,0440
Cholesterol	0,5642
Diabetes	0,9161
Hypertension	0,3212
Previous MI	0,6053
Male gender	0,9880

LV EF = Left ventricular ejection fraction; MI = myocardial infarction

**Table 4:** Multivariate analysis – Hard events (cardiac death + nonfatal AMI + stroke)

			**95% CI**	
**Variable**	**p value**	**HR**	**Lower**	**Upper**

LV EF	<0,01	3,0	1,3	7,1
Aorta plaque	=0,04	2,4	1,0	5,5

HR = Hazard Ratio; CI = Confidence Interval

## Discussion

Atherosclerosis of the descending aorta observed during transesophageal echocardiography is a useful predictor of future cardiovascular events.

### Comparison with previous studies

Our data are in keeping up with previous findings, showing that patients with atherosclerosis of the thoracic aorta have higher probability of coexisting coronary artery disease [2-8]. In those series the positive predictive value of TEE varied between 64% and 95%, whereas the negative predictive value was consistently high (between 82 and 99%.), indicating that in the absence of echocardiographically assessed atherosclerotic plaque in the thoracic aorta the probability of coronary artery disease is unlikely. Furthermore, Khoury et al have demonstrated, that atherosclerotic plaques in patients with coronary artery disease were found predominantly in the descending aorta (in 93%) and in the aortic arch (in 80%), whereas the ascending aorta was the least involved (in 37%) [9]. Atherosclerosis is a complex polygenic, multifactorial vascular disorder associated with many differing and changing metabolic, anatomic and clinical manifestations [13]. The presence of atherosclerotic plaque in the thoracic aorta, as shown by chest x-ray, has been shown in previous studies to be correlated with an increased risk of cardiovascular death [14,15]. However, several studies have also demonstrated that the generation of acute coronary syndromes is not necessarily related to plaque severity rather to its morphology and complexity. From histopathologic and vascular biologic studies [13,16] plaque composition and vulnerability (type of lesion) rather than degree of stenosis (size of lesion) have emerged as crucial factors leading to sudden rupture of the plaque surface, usually with thrombosis superimposed, which underlies the great majority of infarctions. Angiographic studies also suggest that the most frequent situation giving rise to infarction is the occlusion of previously noncritical stenoses [17], which are more prevalent than the possibly more dangerous severe stenoses [18]. Taken together, these studies suggest that in two of three infarctions the culprit lesions had only mild to moderate stenosis on initial evaluation in a substantial number of patients. This is again consistent with our finding that significant coronary artery disease is in close relationship with the atherosclerosis of the aorta but more severe the atherosclerosis is, higher the probability of spontaneous cardiovascular events.

### Clinical implications

One of the most important first steps in stratifying risk among patients with proven or suspected coronary artery disease is the identification of patients at high risk for coronary or vascular events during the course of the next few months or years. To date, left ventricular dysfunction, the number of diseased vessels, and the severity of myocardial ischemia have emerged as important determinants of survival [19]. Our data suggest that atherosclerosis of the descending aorta observed by a simple, routine transesophageal echocardiographic examination can be an additional prognostic marker in identifying patients for higher risk for cardiovascular events. When patients are referred to transesophageal testing for whatever reason, a semiquantitative description of atherosclerotic burden of the descending aorta should be always included in the prognostic stratification.

### Study limitations

This study has several limitations. The study population was highly heterogeneous, reflecting the garden variety of patients referred to the echo lab for transesophageal testing: patients with known or suspected coronary artery disease or cerebrovascular disease coexist with patients with congenital or acquired valvular disease. Therefore, our findings cannot be directly translated into the general population, and further prospective studies are needed at this point to evaluate the prognostic value of transesophageally detected aortic pathology in more sharply defined clinical subsets.

### Future directions

The morphological characterization of atherosclerotic plaque has not been performed in our study, neither in terms of plaque content [20] nor of plaque geometry [21]. Ultrasonic tissue characterization technology can be applied for a more accurate and quantitative description of echocardiographic plaque structure and profile [22]. Both these criteria have documented the prognostic impact in the carotid artery [23]. Thereby, the prognostic value of ultrasonic assessment of aortic atherosclerosis can certainly be further improved with more quantitative, albeit more technologically demanding, image analysis. It is however important, that even a semiquantitative, subjective and extremely simple assessment of atherosclerosis from transesophageal images applied on an extremely heterogeneous population yields powerful prognostic stratification, even when hard prognostic end-points are considered.

## Supplementary Material

Additional File 1 Grade I. atherosclerosis of the descending aorta. Intimal thickening. Click here for file

Additional File 2 Small plaque on the descending aorta, corresponding to Grade 2. atherosclerosis. Click here for file

Additional File 3 Grade 3. atherosclerosis, with large, multiple plaques. Click here for file

Additional File 4 Grade 3. atherosclerosis, plaque with mobile parts. Click here for file
